# Differential Flavonoid and Other Phenolic Accumulations and Antioxidant Activities of *Nymphaea lotus* L. Populations throughout Thailand

**DOI:** 10.3390/molecules27113590

**Published:** 2022-06-02

**Authors:** Duangjai Tungmunnithum, Samantha Drouet, Laurine Garros, Christophe Hano

**Affiliations:** 1 Department of Pharmaceutical Botany, Faculty of Pharmacy, Mahidol University, Bangkok 10400, Thailand; 2 Laboratoire de Biologie des Ligneux et des Grandes Cultures, INRAE USC1328, Campus Eure et Loir, Orleans University, 28000 Chartres, France; samantha.drouet@univ-orleans.fr (S.D.); laurine.garros@univ-orleans.fr (L.G.); 3 Le Studium Institue for Advanced Studies, 1 Rue Dupanloup, 45000 Orléans, France

**Keywords:** flavonoids, polyphenol, *Nymphaea lotus* L., population level, Nymphaeaceae, floristic regions, antioxidant activity

## Abstract

*Nymphaea lotus* L. is a potential plant in the Nymphaeaceae family that is well-recognized as an economic and traditional medicinal plant in Thailand and other countries. Its pharmacological and medicinal effects have been confirmed. However, there is no study going deeper into the population level to examine the phytochemical variation and biological activity of each population that benefits phytopharmaceutical and medical applications using this plant as raw material. This study was intensely conducted to complete this important research gap to investigate the flavonoid profiles from its floral parts, the stamen and perianth, as well as the antioxidant potential of the 13 populations collected from every floristic region by (1) analyzing their flavonoid profiles, including the HPLC analysis, and (2) investigating the antioxidant capacity of these populations using three assays to observe different antioxidant mechanisms. The results indicated that the northeastern and northern regions are the most abundant floristic regions, and flavonoids are the main phytochemical class of both stamen and perianth extracts from *N. lotus.* The stamen offers higher flavonoids and richer antioxidant potential compared with the perianth. This finding is also the first completed report at the population level to describe the significant correlation between the phytochemical profiles in floral parts extracts and the main antioxidant activity, which is mediated by the electron transfer mechanism. The results from the Pearson correlation coefficients between several phytochemicals and different antioxidant assessments highlighted that the antioxidant capability of these extracts is the result of complex phytochemical combinations. The frontier knowledge from these current findings helps to open up doors for phytopharmaceutical industries to discover their preferred populations and floral parts that fit with their targeted products.

## 1. Introduction

*Nymphaea lotus* L. is a species member of the family Nymphaeaceae. This plant species is an aquatic flowering herb [1,2,3,4,5,6] mainly distributed in Asia and Africa, especially in Thailand, China, India, Sri Lanka, Vietnam, Nepal, and Egypt. [7,8,9]. *N. lotus* is also called by its common name, e.g., the Egyptian lotus water lily or lotus [6,7,8,10]. Furthermore, *N. lotus* is also known as its various vernacular names, for example Bau Sai (in the Thai language), Bashneen Abiad (in the Egyptian language), Sulyeon (in the Korean language), etc.

The stolon, petiole, and peduncle of *N. lotus* are consumed as vegetables in various cooked dishes in Egypt, Indonesia, and many Asian countries, whereas the stamen and perianth are used as ingredients of traditional medicines related to circulatory system diseases [7,8,10,11,12,13,14,15,16]. Additionally, the pharmacological and medicinal effects of *N. lotus* were confirmed at the in vivo level, such as anti-diarrhea effects [17], as well as an anxiolytic and antidepressant potential [18]. The acute and sub-chronic toxicity of the *N. lotus* flower extracts was also investigated in vivo using an albino Wistar rat model [19]. The results of this study showed that this flower extract possesses neuroprotective, immune-boosting, and antioxidant activity without neurotoxicity [19].

Many previous studies reported on the antioxidant potential of *N. lotus* extracts that consist of some links with the accumulation of its phytochemical compounds, especially flavonoids [11,20,21]. The DPPH, ABTS, and FRAP in vitro antioxidant assays were used to examine the antioxidant activity of *N. lotus* flower extracts, using hot water for the preparation of the extracts [20], and the results were similar to the antioxidant activity from the synthetic antioxidant BHT (butylated hydroxytoluene). Additionally, *N. lotus* stamen ethanolic extracts showed a similar antioxidant capacity as BHT, which was determined by DPPH, ABTS, and FRAP assays [11]. Moreover, petal ethanolic extracts provided the similar antioxidant trend as ascorbic acid, as determined by the DPPH assay [21]. According to the previous report, a strong correlation between this antioxidant potential and the flavonoid content was observed in *N. lotus* [11,20,21], but there is no report at the plant population level that will help to provide a precise view of flavonoid accumulation and antioxidant activities, which would be helpful for future pharmaceutical and cosmetic applications.

Accordingly, the goals of the current study were (1) to investigate the phytochemical variation, including the phenolics, flavonoids, and anthocyanins, in the floral parts (stamen and perianth), and (2) to determine the antioxidant potentials of the collected 13 *N. lotus* populations across the different localities from each floristic region in Thailand, which is well-known as the hotspot of plant diversity. We aimed to complete this knowledge by examining the total phenolic, anthocyanin, and flavonoid (including HPLC determination of the main flavonoids) contents, as well as the antioxidant capacity using three different in vitro antioxidant approaches (ORAC, DPPH, and FRAP) based on the major different mechanisms. The Pearson correlation coefficients (PCCs) between several phytochemical classes and different antioxidant assays were also employed for the additional understanding of the relationship between these variables.

## 2. Results and Discussion

### 2.1. N. lotus Populations and Botanical Description

**Figure 1:**
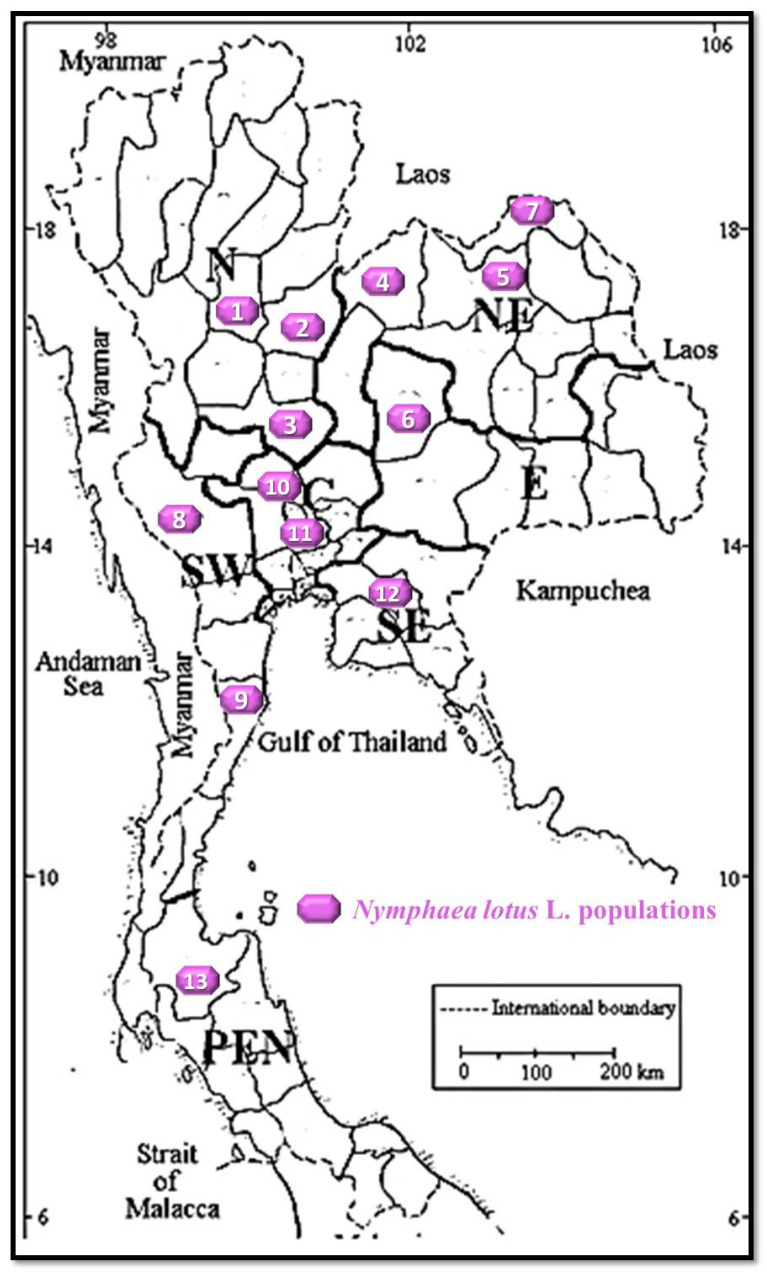
The distribution map of *N. lotus* populations collected from their natural habitats from every floristic region of Thailand. The numbers 1–13 on the distribution map indicate the population number.

**Figure 2:**
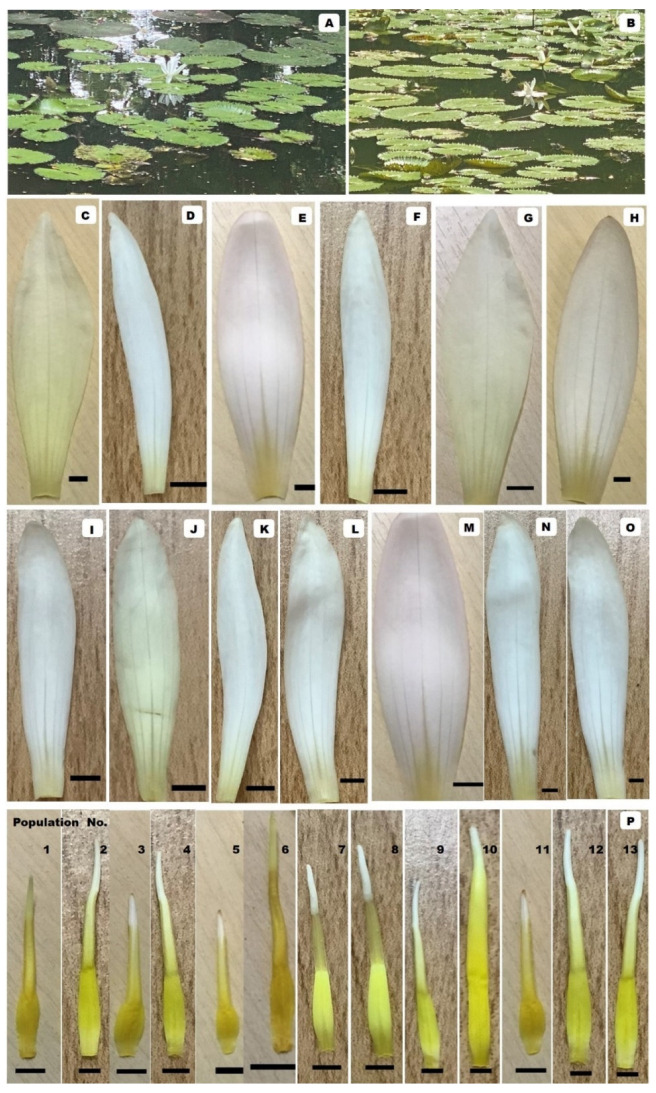
*N. lotus*: (**A**,**B**) natural habitats; (**C**–**O**) perianths of Population No. 1–13, respectively; (**P**) stamens of Population No. 1–13, respectively; bar scale = 0.5 cm. Photos were taken in Thailand by Duangjai Tungmunnithum.

According to this field study and intense search for *N. lotus* living specimens from its natural habitats, the 13 populations of *N. lotus* were collected from various provinces (localities), covering all the floristic regions of the country (Figure 1). The details of each collected population are provided in Table 1, as well as the photo of *N. lotus*’s stamen and perianth from each population (Figure 2).

The distribution map of the 13 collected *N. lotus* populations throughout Thailand is provided in Figure 1. According to the distribution of these 13 populations of this plant species (Table 1 and Figure 1), the most abundant floristic region in Thailand is the northeastern and northern floristic regions, where 3 populations of *N. lotus* were equally found. The botanical description of *N. lotus* (the Nymphaeaceae family) used in this study is provided in the paragraph below.

**Species description**: Aquatic perennial herb. Rhizome erect, many slender stolons. Leaf suborbicular or ovate-elliptic, margin dentate with acute teeth; 16–55 cm; adaxial glabrous, green or dark green; abaxial pubescent, dark purple; base cordate. Petiole slender 1.8–7.5 m long. Flower simple, emergent; outer perianth oblong or narrowly oblong, dark green, conspicuously veined, 5–7 cm long; inner perianth oblong or narrowly oblong, white, creamy white, red, or pale pink, 3.5–8.5 cm; stamen numerous, filament almost equal to anther in length, connective apically unappendaged. Pistil one, carpel many and united, ovary half-inferior, parietal placentation. Fruit ovoid, 2.8–5.3 cm. Seed ellipsoid, 1–2 mm, many longitudinal ridges on seed surface.

**Specimens examined**: Perianths and stamens of *N. lotus* populations no. 1–13.

**Synonyms**: *Nymphaea lotus* var. *rogeonii* A.Chev.; *Nymphaea liberiensis* A.Chev.; *Castalia edulis* Salisb.; *Castalia lotus* Tratt.; *Castalia pubescens* Blume; *Castalia pubescens* Wood; *Castalia mystica* Salisb.; *Castalia sacra* Salisb.; *Nymphaea dentata* Schumach. & Thonn.

**Flowering season**: starting from July to December.

### 2.2. Phytochemical Characterization of the 13 N. lotus Populations

The total phenolic contents (TPCs), total flavonoid contents (TFCs), and monomeric anthocyanin contents (MACs) found in the stamen (S) and perianth (P) extracts of the 13 *N. lotus* populations (Table 2) from all of Thailand’s floristic regions ranged from single to double or even triple for the TPC of the stamen extracts, which illustrated the high heterogeneity in the accumulations of phenolics/polyphenols for these floral parts of this aquatic medicinal plant. Consequently, it is important to evaluate these contents prior to their use for further studies, such as to determine or compare the biological activity of these different floral parts (perianth and stamen) and/or phytopharmaceutical/cosmeceutical applications.

The TPC ranged from 301.6 (NLs#8) to 619.2 (NLs#3) mg/100 g DW in gallic acid equivalent for the stamen extracts and from 204.6 (NLs#2) to 288.2 (NLs#8) mg/100 g DW in gallic acid equivalent for the perianth extracts. The TFC ranged from 475.0 (NLs#2) to 711.6 (NLs#11) mg/100 g DW in quercetin equivalent for the stamen extracts and from 303.0 (NLp#2) to 415.9 (NLp#1) mg/100 g DW in quercetin equivalent for the perianth extracts. The MAC ranged from 1.2 (NLs#9) to 3.7 (NLs#3) mg/100 g DW in cyanidin-3-O-glucoside equivalent for the stamen extracts and from 1.4 (NLp#8) to 2.2 (NLp#1) mg/100 g DW in cyanidin-3-O-glucoside equivalent for the perianth extracts. According to these results, the stamen is the raw plant material that provides high TPC, TFC, and MAC compared with that of the perianth.

**Figure 3:**
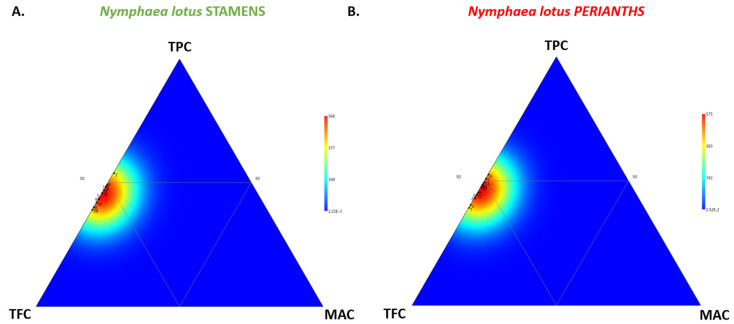
Ternary plot showing the visualization of the relative proportion of TPC, TFC, and MAC within the stamen (**A**) and perianth (**B**) extracts of 13 *N. lotus* populations originating from various floristic regions in Thailand.

The results from the ternary plot displaying the visualization of the relative proportion of TPC, TFC, and MAC within the stamen and perianth extracts (Figure 3) indicated the significance of TFC as a key factor for phytochemical variation. The heatmap distribution illustrates the marked shift of the first bottom triangle (high TFC) for each floral part, such as the stamen and perianth of *N. lotus*.

A few studies compared the TPC or TFC in the different floral parts, e.g., the stamen and perianth of *N. lotus* [9,11,12,20,22]. In addition, there is no previous report on the flavonoid phytochemical profiles at the population level of this plant species. This present study served as the first investigation dealing with both the TPC and TFC, as well as the MAC of *N. lotus*, comparing their natural populations from various localities throughout the whole country. The results from this current study also support the finding of previous works that the accumulation of these phytochemicals varies depending on the floral organ/flower parts [11,12,13,19,20,22]. Interestingly, this study also provided the first report on the significant correlation between the phytochemical contents, especially for the TFCs in each floral part: the perianth and stamen extracts for each phytochemical class (Figure S1 in Supplementary Materials).

According to these results, it is clearly seen that the polyphenol accumulation capacity of the different populations in the same species of this *Nymphaea* genus are varied, comparing between each floral part. For example, the highest TFC (Table 2) in the stamen belonged to population 11 (NLs#11) as 711.6 mg/100 g DW in quercetin equivalent, whereas the highest TFC in the perianth belonged to population 1 (NLp#1) as 415.9 mg/100 g DW in quercetin equivalent. This provides alternative choices for the future phytopharmaceutical/phytocosmeceutical applications, in which the industrial sectors are able to select their raw material from their preferred populations based on the needed polyphenol phytochemical profile [6,23,24,25,26,27]. Consequently, it is also intriguing to note the relative impacts of environmental factors (e.g., climate, nutrients, geography, and so forth) and genetic backgrounds that may possibly influence the variation of these polyphenol phytochemical compounds [28,29,30] on the population level. Therefore, it would be very interesting to assess and evaluate these factors over the multiple years of the specific *N. lotus* population as well as on the same study site/locality in the future. In addition, the in vitro culture of descendant plants from these populations would be a fascinating perspective to be evaluated.

**Figure 4:**
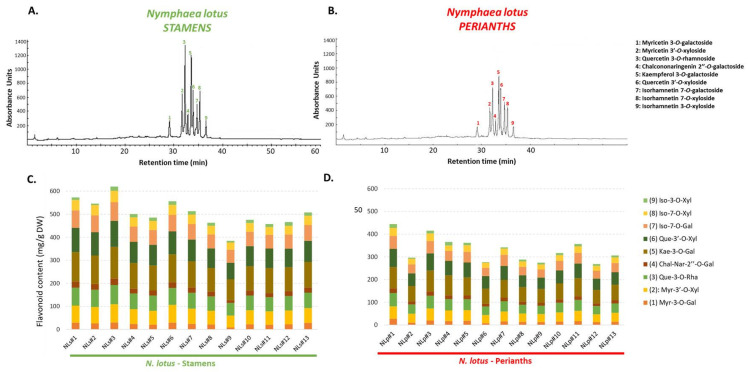
HPLC chromatograms (recorded at 320 nm) of the stamen (**A**) and perianth (**B**) extracts of 13 *N. lotus* populations originating from various floristic regions in Thailand. Absolute quantification (mean of three independent replicates) of the main flavonoids in the stamen (**C**) and perianth (**D**) extracts of 13 *N. lotus* populations originating from various floristic regions in Thailand.

According to these results, it is clearly identified that flavonoids are the major phytochemical class of *N. lotus* stamen and perianth extracts and play the key role in contributing to the obtained variations. Additionally, high-performance liquid chromatography (HPLC) analyses were performed to provide a more comprehensive understanding in terms of qualitative and quantitative changes in both floral parts (stamen and perianth extracts) (Figure 4, Table S1).

The results from the HPLC analyses (Figure 4, Table S1) indicated that the flavonoid concentrations in the floral parts of *N. lotus* extracts ranged between 3.1 (isorhamnetin 3-O-xyloside, NLp#6) and 137.3 (kaempferol 3-O-galactoside, NLs#3) mg/100 g DW. These current analyses clearly confirmed that *N. lotus* stamen extracts are the richer raw plant material in flavonoids than perianth extracts. Comparing the flavonoid content at the population level, the *N. lotus* stamen extracts were quite balanced, and the prominent flavonoids (Figure 4, Table S1) consisted of kaempferol 3-O-galactoside, quercetin 3′-O-xyloside, quercetin 3-O-rhamnoside, isorhamnetin 7-O-galactoside, and myricetin 3′-O-xyloside. The perianth extracts from *N. lotus* provided the similar trend of prominent flavonoids, with a lower amount of flavonoid content (Figure 4, Table S1) when compared with that of the stamen extracts. The accumulated flavonoids were mostly kaempferol 3-O-galactoside, quercetin 3′-O-xyloside, quercetin 3-O-rhamnoside, isorhamnetin 7-O-galactoside, and myricetin 3′-O-xyloside. In comparison, in the previous works that investigated a single population of *N. lotus* in Thailand, kaempferol 3-O-galactoside and quercetin 3′-O-xyloside were reported as the main flavonoid bioactive compounds, as well [9,11]. Compared with the other previous research conducted in the Asian region using the plant samples from the same Nymphaeaceae family, the obtained flavonoids from our findings were also reported in the previous studies of Zhu et al. [22] and Yin et al. [20]. However, these previous works investigated different plant cultivars in the same genus without reports on the identified plant material [20,22] at the species level, so the deeper details, such as the TPC, TFC, or the flavonoid concentrations from HPLC analysis, cannot be compared. Thus, this current study offered the first report on the quantification of the different flavonoids accumulated in both stamen and perianth extracts of this medicinal plant species inside its populations, collected from every floristic region of Thailand, which is a well-known hotspot of potential medicinal plants.

**Figure 5:**
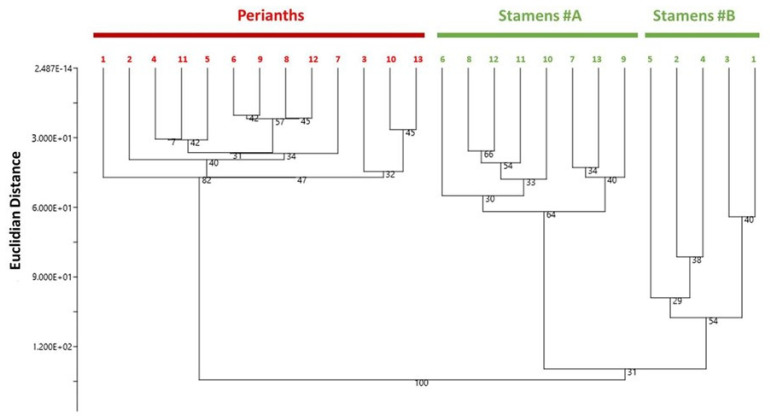
Hierarchical clustering analysis dendrogram according to the phytochemical composition of the stamen and perianth extracts of 13 *N. lotus* populations originating from various floristic regions in Thailand. The percentages of replicate trees in which associated samples clustered together in the bootstrap test (percentage of 5000 replicates) are indicated next to the branches.

Then, the hierarchical clustering analysis (HCA) was also employed to identify the potential groupings between diverse *N. lotus* samples from various populations (Figure 5).

The results from the HCA analysis showed that the clustering occurred primarily at the floral-parts level based on their phytochemical profiles. On the one hand, the *N. lotus* perianth extracts were clustered together in the same group (Figure 5, Perianths). On the other hand, the *N. lotus* stamen extracts were clustered into two subgroups (Figure 5, Stamens#A and Stamens#B) based on their different flavonoid concentrations, the first subgroups (Figure 5, Stamens#B), comprising of populations #1–5, having richer flavonoids compared with the second subgroups (Figure 5, Stamens#A), consisting of populations #6–13. Nevertheless, no discernable pattern exists to demonstrate the significant genetic background factors. In view of the wide geographic distribution of *N. lotus* populations across the different floristic areas of the country, environmental factors may possibly be the explanation for at least some parts of the heterogeneity in the phytochemical profiles that were observed in these studied populations.

Altogether, these obtained results offer a complete image of flavonoid phytochemical profiles, especially the wide variations that were observed at the organs/floral-parts level in different *N. lotus* populations covering all the floristic regions throughout Thailand. It may be possible to anticipate that these phytochemical variations may affect the pharmacological/biological effects of this medicinal plant species. Therefore, we examined the antioxidant capability of these *N. lotus* extracts from both floral parts. Flavonoid phytochemical compounds have been proven to have various health benefits through antioxidant activity [25,31].

### 2.3. Antioxidant Activity

The *N. lotus* antioxidant potentials of both floral parts (stamen and perianth) extracts from different natural populations across all the floristic regions to scavenge the free radicals were evaluated by two different major antioxidant mechanisms, such as (1) the hydrogen atom transfer (HAT) mechanism, determined by the ORAC assay, and (2) the single electron transfer (SET) mechanism, evaluated with the FRAP assay, while the DPPH assay was used to evaluate both mechanisms [32,33]. The results of antioxidant potentials from the *N. lotus* populations determined by these three antioxidant tests (ORAC, FRAP, and DPPH) are shown in Table 3.

**Figure 6:**
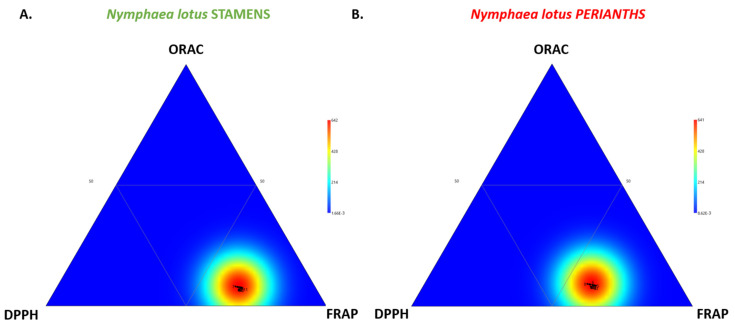
Ternary plot showing the visualization of the relative contribution of the different in vitro assays (ORAC, DPPH, and FRAP) to the antioxidant capacity of the stamen (**A**) and perianth (**B**) extracts of 13 *N. lotus* populations originating from various floristic regions in Thailand.

According to the in vitro cell-free antioxidant activity of the stamen (NLs) and perianth (NLp) extracts (Table 3), the oxygen radical antioxidant capacity (ORAC) ranged from 24.0 (NLs#9) to 35.9 (NLs#3) µmol TEAC/g DW for the *N. lotus* stamen extracts and from 23.1 (NLp#13) to 26.8 (NLp#1) µmol TEAC/g DW for that of the perianth extracts. The DPPH free radical scavenging activity ranged from 85.3 (NLs#9) to 124.4 (NLs#3) µmol TEAC/g DW for the *N. lotus* stamen extracts and from 80.2 (NLp#11) to 94.6 (NLp#1) µmol TEAC/g DW for that of the perianth extracts. The FRAP reducing power ranged from 201.4 (NLs#13) to 304.0 (NLs#3) µmol TEAC/g DW for the *N. lotus* stamen extracts and from 150.7 (NLs#8) to 188.2 (NLp#1) µmol TEAC/g DW for that of the perianth extracts. These present results indicated that the SET may play a more important role in the antioxidant mechanism of *N. lotus* than the HAT mechanism (Figure 6), with the FRAP assay for both floral parts (stamen extracts in Figure 6A and perianth extracts in Figure 6B) contributing the most to their antioxidant capacity.

The results of this current work are consistent with the previous works [11] on *N. lotus* stamen ethanolic extract. Its antioxidant mechanism was evaluated using DPPH, ABTS, and FRAP assays. The previous work [11] showed that the obtained *N. lotus* stamen ethanolic extract offered a similar antioxidant capacity as Butylated hydroxytoluene (BHT), which is a synthetic antioxidant, and suggested that the main antioxidant mechanism is linked to the electron transfer mechanism. Another previous study from Semaming et al. [21] focused on petal or perianth ethanolic extracts, which also indicated a similar trend of antioxidant flavonoids. In addition, Yin et al. [20] investigated the in vitro antioxidant potential of flower hot water extracts from various cultivars of plants in the same genus *Nymphaea* by using DPPH, ABTS, and FRAP assays; the results from this study showed the same trend of synthetic antioxidant BHT. Both this present study and the previous works pointed out the strong correlation between flavonoid content and the antioxidant capacity of *N. lotus* [11,20,21]. According to the obtained results, the major antioxidant mechanism of the floral parts of *N. lotus* is mediated by the electron transfer mechanism; this may link to the position and degree of hydroxylation and methoxylation of the flavonoid ring B [6,34]. Interestingly, our present study, which is the first report conducted at the population level from various floristic regions/localities of this medicinal plant species, provided the viewpoints of flavonoid phytochemical profiles and their contribution to the antioxidant mechanism.

### 2.4. Correlation Analysis

**Figure 7:**
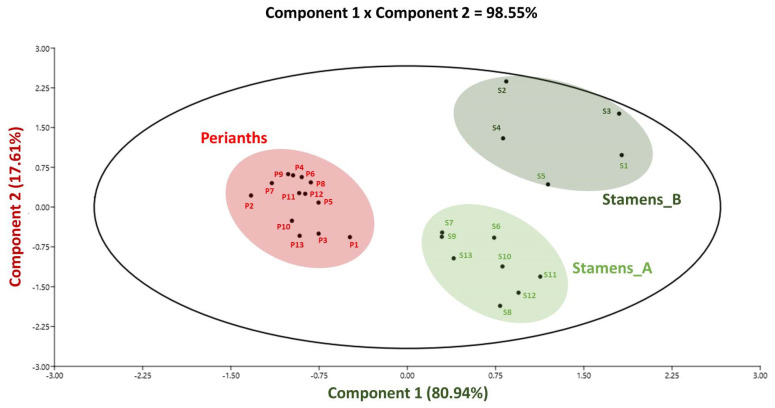
Principal component analysis (PCA) linking the phytochemical profile and antioxidant capacity of the stamen and perianth extracts of 13 *N. lotus* populations originating from various floristic regions in Thailand. Variance of component 1 = 80.94%, and component = 17.61%. S: stamen extract; P: perianth extract; each number in blue represents the different populations. The corresponding loading score plots for components 1 and 2 are presented in Figure S1.

According to the principal component analysis (PCA), various variables were used to determine the relevant connections between the antioxidant potential and metabolic composition within the floral parts: the stamen and perianth extracts from *N. lotus* populations collected from different localities/floristic regions covering the whole country. (Figure 7).

The biplot generated from the PCA analysis in Figure 7 explained 98.55% of the initial variability (Figure 5). The TFC and the FRAP antioxidant assay were the key contributing factors to the discrimination along the component 1 axis, which accounted for 80.94% of the initial variability (Figure S1), whereas the second component axis accounted for only 17.61%. According to the results from this PCA analysis, the two different major clusters of the stamen and perianth were shown to be significantly different from one another based on their TFC and FRAP antioxidant activity. The stamen clusters were divided into two subclusters, such as stamen_A, consisting of population #1–5 (Figure 7, stamen_A (S6–S13)), and stamen_B, consisting of population #1–5 (Figure 7, stamen_A (S1–S5)). Importantly, these clusters illustrated that *N. lotus* stamen extracts are higher in flavonoids and have a higher FRAP antioxidant capacity than that of perianth extracts. For the stamen clusters, subcluster stamen_B offered richer sources of flavonoids and FRAP antioxidant activity compared with that of subcluster stamen_A. Our present study verified the value of stamen as the potential raw plant material for phytopharmaceutical and cosmeceutical applications, based on the antioxidant flavonoids derived from *N. lotus*.

Pearson correlation coefficients (PCCs) were calculated to investigate the relationship between each phytochemical compound and antioxidant capacity in Table 4.

The results from the Pearson correlation coefficients (PCCs) clearly illustrated the strong association between TFCs, including each individual flavonoid and various antioxidant assays (ORAC, DPPH, and FRAP). The significant correlations between several phytochemical classes and diverse antioxidant assays were distinguished. This emphasized that the complex phytochemical combinations affect the antioxidant capacity of *N. lotus* extracts more than that of a single molecule [35]. To consider the key role of electron transfer antioxidant mechanism in contributing to the antioxidant potential of *N. lotus* extracts, the strongest and most significant correlations linked TFC and myricetin 3′-O-xyloside, quercetin 3-O-rhamnoside, kaempferol 3-O-galactoside, quercetin 3′-O-xyloside, isorhamnetin 7-O-galactoside, and isorhamnetin 7-O-xyloside.

## 3. Materials and Methods

### 3.1. Chemicals and Reagents

The reagents as well as the solvents for extraction and HPLC analysis that were used in this study were analytical grade or the highest available purity reagents/solvents (Thermo Fischer Scientific, Illkirch, France). The Milli-Q water-purification system (Merck Millipore Fontenay sous Bois, Paris, France) was employed to purify deionized water. Every prepared solution was filtered through 0.45 µm of nylon syringe membranes prior to use in HPLC analysis. All the standard compounds were purchased from Extrasynthese (Genay, France).

### 3.2. Plant Materials

Living *N. lotus* plants were searched and collected from their natural habitats, covering all the floristic regions in Thailand, such as the northern (N), northeastern (NE), central (C), southeastern (SE), eastern (E), southwestern (SW), and peninsula (PEN) regions. After the literatures review and the herbarium specimen study of *N. lotus*, in order to obtain the fundamental information about the distribution, the targeted populations from various localities from every floristic region in Thailand were searched, so as to seek for *N. lotus* living specimens from the fields. The collected *N. lotus* samples were identified at the species level by using the taxonomic key and description in the existing Floras [1,2,3,4,5], as well as compared with the herbarium specimens kept in Forest Herbarium (BKF), Bangkok, Thailand, The Prof. Kasin Suvatabandhu Herbarium, Chulalongkorn University, (BCU). Herbarium abbreviations are used according to Thiers [36]. After that, the stamens and perianths from 13 populations of *N. lotus* were air-dried and then prepared following the World Health Organization recommendations [37].

### 3.3. Extraction

A 100 mg/sample of dried stamen or perianth of *N. lotus* was placed into the 5 mL quartz tubes, which were equipped with the vapor condenser, and was then used for ultrasound-assisted extraction in the 1 mL 90% (*v*/*v*) aqEtOH in USC1200TH ultrasonic bath (Prolabo, Fontenay-sous-Bois, France) using the optimized extraction conditions (30 kHz frequency at 45 °C for 45 min) [11]. After that, the extracts were centrifuged at 5000× *g* for 15 min (Heraeus Biofuge Stratos, Thermo Scientific, Illkirch, France). Then, the supernatant obtained from this step was filtered by using a 0.45 μm of nylon syringe membrane (Merck Millipore, Saint-Quentin Fallavier, France). Then, flavonoid enrichment was performed through the additional DAX-8 (Merck Millipore, Saint-Quentin Fallavier, France) macroporous resin purification process that was previously described by Tungmunnithum et al. [11].

### 3.4. Determination of the Total Phenolic Content (TPC)

The TPC was determined using the Folin–Ciocalteu protocol as well as microplate spectrophotometry, following the previous study in 2020 [38]. The absorbance was measured at 650 nm by using a spectrophotometer (BioTek ELX800 Absorbance Microplate Reader, BioTek Instruments, Colmar, France). The standard curve (0–40 µg/mL; R^2^ = 0.998) of gallic acid (Merck, Saint-Quentin Fallavier, France) was then performed, so as to express the total phenolic content in mg of the gallic acid equivalents/g DW (mg GAE/100 g dry weight (DW)).

### 3.5. Determination of the Total Flavonoid Content (TFC)

The TFC was determined using the colorimetric aluminum trichloride (AlCl_3_) method, as previously described by Tungmunnithum et al. [11]. Then, the 200 µL of mixture was obtained in a microplate by using 20 µL of the extract, 10 µL of AlCl_3_ (10% (*w*/*v*)), 10 µL of potassium acetate 1 M, and 160 µL of deionized water. After that, a microplate reader (Multiskan GO, Thermo Fischer Scientific, Illkirch, France) was used to investigate the absorbance at 415 nm after the 30 min of incubation time at 25 °C in the dark. Then, the TFC was expressed in mg/100 g dry weight (DW) of the quercetin equivalent by using a five-point calibration line (linearity range from 0 to 40 µg/mL of quercetin concentration with R^2^ of 0.998).

### 3.6. Determination of the Total Monomeric Anthocyanin Content (MAC)

The TAC was measured following the colorimetric method, as suggested in the previous study of Wrolstad [39]. The absorbance was determined by using a spectrophotometer (BioTek ELX800 Absorbance Microplate Reader, BioTek Instruments, Colmar, France) at 510 and 700 nm. After that, the standard curve (0–100 µg/mL, R^2^ = 0.999) of cyanidin-3-O-glucoside (Merck, Saint-Quentin Fallavier, France) was used to express the content of total anthocyanin in mg of the cyanidin-3-*O*-glucoside equivalents/g DW (mg CAE/100 g DW).

### 3.7. High-Performance Liquid Chromatography (HPLC)

For the HPLC analysis, the high-performance liquid chromatography system consisting of an autosampler, Varian (Les Ulis, France) Prostar 230 pump, and Varian Prostar 335 photodiode array detector, was employed to analyze and was controlled by the Galaxie software (v1.9.3.2, Varian, Les Ulis, France). Then, the separation was carried out at 40 °C using a Purospher RP-18 column (250 × 4.0 mm internal diameter; 5 µm) (Merck Chemicals, Molsheim, France). The mobile phase contained a mixture of methanol (solvent A) as well as the HPLC grade water (solvent B), which were acidified by 0.05% formic acid. For the next steps, the linear gradient was applied to the mobile phase variation from a 5:95 (*v*/*v*) to 100:0 (*v*/*v*) mixture of solvents A and B, respectively, using a flow rate of 1.30 mL per min. The injection volume was 3 µL, and the maximum back pressure was 110 bar. The detection was performed at 320 nm. Flavonoid phytochemical compounds were identified by comparing them with the purchased authentic standards (Sigma Aldrich, Saint Quentin Fallavier, France).

### 3.8. The In Vitro Cell-Free Antioxidant Assays

The 3 in vitro cell-free antioxidant assays, the DPPH (2,2-diphenyl-1-picrylhydrazyl), FRAP (Ferric Reducing Antioxidant Power), and ORAC (oxygen radical antioxidant capacity) assays, were employed in this study to examine the antioxidant potential of the obtained extracts, following the protocols adapted to a microplate reader (VICTOR Nivo 5, PerkinElmer, Villebon-sur-Yvette, France), as described in the previous works [11,28].

### 3.9. Statistical Analysis

The statistical analyses were performed by using XLSTAT 2019 suite (Addinsoft, Paris, France) as well as PAST4.0 [40]. The data composed of at least 3 independent replicates were presented as the means and standard deviations. A Student’s t-test was performed for the statistical comparative analysis. The significant differences at *p* < 0.05, 0.01, and 0.001 were noted using *, **, and ***, correspondingly. The different letters were used to show significant thresholds at *p* < 0.05.

## 4. Conclusions

To sum up, the collected 13 *N. lotus* natural populations from various floristic regions in Thailand exhibited the high heterogeneity in their polyphenol accumulations, especially in terms of flavonoid phytochemical profiles detected in their floral parts, both perianths and stamens. The results from this present analysis also indicated that flavonoids are the key phytochemical class of these extracts, and the stamen of *N. lotus* provides richer flavonoid phytochemical compounds than that of the perianth. Remarkably, this study is also the first report conducted at the population level of *N. lotus* and describing the significant correlation between the phytochemical profiles in perianth and stamen extracts and the antioxidant capacity. Furthermore, the three in vitro cell-free antioxidant approaches (ORAC, DPPH, and FRAP) revealed that the antioxidant potential of the observed stamen and perianth from the 13 *N. lotus* populations was mainly mediated by the electron transfer mechanism, which may possibly be the result of the complex phytochemicals rather than any single bioactive molecule. Our finding is the frontier knowledge focusing on *N. lotus*’s phytochemical diversity, particularly the flavonoids phytochemical class and antioxidant activity from their floral parts at the population level, which will help to open up doors of potential raw plant materials for phytocosmeceutical or other phytopharmaceutical industries to choose their preferred *N. lotus* populations for their future product development.

## Figures and Tables

**Table 1:** The collected 13 *N. lotus* populations throughout Thailand.

Population No.	Collected Localities/Provinces	Floristic Regions	Collected Time		Plant Parts Used in This Study	
			Months	Seasons	Perianth	Stamen
1	Sukhothai	Northern (N)	April	Summer	√	√
2	Phitsanulok	Northern (N)	August	Rainy	√	√
3	Nakhon Sawan	Northern (N)	September	Rainy	√	√
4	Nong Bua Lam Phu	Northeastern (NE)	May	Summer	√	√
5	Udon Thani	Northeastern (NE)	November	Winter	√	√
6	Chaiyaphum	Eastern (E)	April	Summer	√	√
7	Nong Khai	Northeastern (NE)	September	Rainy	√	√
8	Kanchanaburi	Southwestern (SW)	April	Summer	√	√
9	Prachuap Khiri Khan	Southwestern (SW)	September	Rainy	√	√
10	Sing Buri	Central (C)	November	Winter	√	√
11	Phra NakhonSi Ayuthaya	Central (C)	May	Summer	√	√
12	Chachoengsao	Southeastern (SE)	April	Summer	√	√
13	Phatthalung	Peninsular (PEN)	September	Rainy	√	√

**Table 2:** Phytochemical profiles of stamen (NLs) and perianth (NLp) extracts from 13 *N. lotus* populations originating from various floristic regions in Thailand.

Sample	TPC (mg/100 g DW)	TFC (mg/100 g DW)	MAC (mg/100 g DW)
NLs#1	578.3 ± 22.2 ^b^	680.9 ± 36.2 ^abc^	3.2 ± 0.1 ^b^
NLs#2	556.4 ± 0.5 ^b^	475.0 ± 28.3 ^g^	2.9 ± 0.2 ^b^
NLs#3	619.2 ± 6.2 ^a^	634.7 ± 76.9 ^abc^	3.7 ± 0.2 ^a^
NLs#4	503.1 ± 1.9 ^c^	532.3 ± 6.2 ^f^	1.9 ± 0.1 ^d^
NLs#5	490.3 ± 12.2 ^c^	630.2 ± 32.4 ^cd^	2.3 ± 0.1 ^c^
NLs#6	364.7 ± 32.4 ^d^	614.0 ± 17.6 ^d^	2.0 ± 0.1 ^d^
NLs#7	325.0 ± 30.0 ^de^	550.6 ± 39.0 ^def^	1.9 ± 0.1 ^d^
NLs#8	301.6 ± 21.7 ^def^	693.3 ± 0.3 ^b^	1.3 ± 0.1 ^gh^
NLs#9	330.6 ± 13.4 ^d^	560.9 ± 14.1 ^e^	1.2 ± 0.1 ^h^
NLs#10	349.5 ± 24.1 ^d^	657.1 ± 3.4 ^c^	1.3 ± 0.1 ^gh^
NLs#11	374.3 ± 46.2 ^d^	711.6 ± 9.7 ^a^	1.5 ± 0.1 ^defg^
NLs#12	335.7 ± 23.5 ^d^	702.3 ± 2.4 ^a^	1.5 ± 0.2 ^defg^
NLs#13	311.2 ± 30.0 ^def^	590.6 ± 16.2 ^de^	1.3 ± 0.1 ^h^
NLp#1	239.3 ± 10.8 ^h^	451.9 ± 15.9 ^g^	2.2 ± 0.2 ^cd^
NLp#2	204.6 ± 30.3 ^h^	303.0 ± 14.8 ^k^	1.5 ± 0.1 ^defg^
NLp#3	215.4 ± 54.9 ^gh^	413.0 ± 51.0 ^ghi^	1.7 ± 0.2 ^def^
NLp#4	266.7 ± 56.3 ^defgh^	329.5 ± 26.2 ^hij^	1.6 ± 0.1 ^def^
NLp#5	259.8 ± 37.7 ^efgh^	386.4 ± 28.6 ^hi^	1.6 ± 0.2 ^de^
NLp#6	278.3 ± 22.7 ^efg^	345.9 ± 51.5 ^hi^	1.8 ± 0.3 ^def^
NLp#7	238.2 ± 64.3 ^efg^	313.4 ± 0.7 ^k^	1.5 ± 0.2 ^fg^
NLp#8	288.2 ± 14.8 ^efg^	363.2 ± 3.6 ^i^	1.4 ± 0.0 ^g^
NLp#9	274.6 ± 24.1 ^efg^	328.7 ± 12.2 ^jk^	1.5 ± 0.1 ^ef^
NLp#10	217.0 ± 5.5 ^h^	375.7 ± 14.7 ^hi^	1.6 ± 0.0 ^f^
NLp#11	256.8 ± 22.4 ^fgh^	355.7 ± 45.0 ^hij^	1.8 ± 0.1 ^de^
NLp#12	268.6 ± 0.8 ^g^	368.3 ± 26.7 ^hij^	1.5 ± 0.1 ^defg^
NLp#13	206.5 ± 62.6 ^fgh^	399.7 ± 11.1 ^h^	1.7 ± 0.1 ^def^

NL: *Nymphea lotus* extract; s: stamen; p: perianth; #i indicates the population number i; TPC: total phenolic content; TFC: total flavonoid content; MAC: total monomeric anthocyanin content; DW: dry weight. Different superscript letters indicate significant differences at *p* < 0.05.

**Table 3:** In vitro cell-free antioxidant activity of stamen (NLs) and perianth (NLp) extracts of 13 *N. lotus* populations collected from various floristic regions in Thailand.

Sample	ORAC (µmol TEAC)	DPPH (µmol TEAC)	FRAP (µmol TEAC)
NLs#1	33.1 ± 0.4 ^b^	115.1 ± 1.3 ^b^	288.5 ± 3.1 ^b^
NLs#2	31.9 ± 0.4 ^c^	111.3 ± 1.3 ^c^	279.4 ± 10.7 ^b^
NLs#3	35.9 ± 1.2 ^a^	124.4 ± 4.0 ^a^	304.0 ± 3.0 ^a^
NLs#4	27.4 ± 0.4 ^de^	96.4 ± 1.3 ^d^	224.0 ± 1.3 ^c^
NLs#5	28.0 ± 0.4 ^d^	98.3 ± 1.3 ^d^	231.8 ± 0.6 ^c^
NLs#6	27.7 ± 0.1 ^d^	97.4 ± 0.1 ^d^	233.7 ± 7.0 ^c^
NLs#7	27.7 ± 0.8 ^de^	96.5 ± 2.6 ^d^	218.6 ± 0.4 ^d^
NLs#8	24.6 ± 0.4 ^g^	87.1 ± 1.3 ^fg^	203.7 ± 2.4 ^f^
NLs#9	24.0 ± 0.4 ^gh^	85.3 ± 1.3 ^fg^	208.8 ± 3.9 ^ef^
NLs#10	25.7 ± 0.4 ^f^	90.8 ± 1.3 ^ef^	211.3 ± 1.2 ^e^
NLs#11	24.3 ± 0.8 ^fg^	86.2 ± 2.6 ^fgh^	218.9 ± 2.4 ^d^
NLs#12	25.1 ± 0.4 ^fg^	89.0 ± 1.3 ^f^	206.9 ± 1.8 ^ef^
NLs#13	24.6 ± 0.4 ^g^	87.1 ± 1.3 ^fg^	201.4 ± 1.0 ^f^
NLp#1	26.8 ± 1.2 ^ef^	94.6 ± 4.0 ^de^	188.2 ± 0.5 ^g^
NLp#2	24.3 ± 0.1 ^g^	86.2 ± 0.2 ^g^	169.8 ± 2.1 ^h^
NLp#3	25.1 ± 0.4 ^fg^	89.0 ± 1.3 ^f^	181.6 ± 5.3 ^gh^
NLp#4	26.3 ± 0.4 ^ef^	87.5 ± 1.9 ^fg^	174.9 ± 1.5 ^h^
NLp#5	24.1 ± 0.4 ^gh^	85.3 ± 1.6 ^fg^	173.8 ± 1.8 ^h^
NLp#6	25.7 ± 2.0 ^efg^	90.8 ± 6.6 ^def^	174.4 ± 4.8 ^h^
NLp#7	24.6 ± 0.4 ^g^	87.1 ± 1.2 ^fg^	169.9 ± 2.3 ^h^
NLp#8	24.0 ± 0.4 ^gh^	82.5 ± 1.3 ^h^	150.7 ± 0.3 ^j^
NLp#9	23.7 ± 0.8 ^gh^	81.3 ± 2.5 ^h^	156.9 ± 3.6 ^ij^
NLp#10	24.6 ± 0.4 ^fg^	81.7 ± 1.6 ^h^	159.0 ± 1.5 ^i^
NLp#11	24.2 ± 0.1 ^g^	80.2 ± 3.1 ^h^	156.8 ± 1.6 ^i^
NLp#12	23.4 ± 0.4 ^h^	81.3 ± 2.7 ^h^	154.9 ± 3.3 ^ij^
NLp#13	23.1 ± 0.4 ^h^	81.5 ± 3.1 ^h^	160.7 ± 4.3 ^ij^

NL: *N. lotus* extract; s: stamen; p: perianth; #i indicates the population number i; ORAC: oxygen radical absorbance capacity; DPPH: 2,2-diphenyl-1-picrylhydrazyl; FRAP: ferric reducing antioxidant power; TEAC: Trolox-C equivalent antioxidant capacity. Different superscript letters indicate significant differences at *p* < 0.05.

**Table 4:** Pearson coefficient correlation between phytochemical profiles and antioxidant activities of the stamen and perianth extracts from 13 *N. lotus* populations.

Compound	ORAC	DPPH	FRAP
Myricetin 3-O-galactoside	0.669 **	0.670 **	0.727 ***
Myricetin 3′-O-xyloside	0.755 **	0.755 **	0.910 ***
Quercetin 3-O-rhamnoside	0.770 **	0.770 **	0.921 ***
Chalcononaringenin 2′′-O-galactoside	0.796 **	0.796 **	0.870 ***
Kaempferol 3-O-galactoside	0.763 **	0.763 ***	0.924 ***
Quercetin 3′-O-xyloside	0.782 **	0.783 ***	0.928 ***
Isorhamnetin 7-O-galactoside	0.751 **	0.751 ***	0.915 ***
Isorhamnetin 7-O-xyloside	0.770 **	0.770 ***	0.907 ***
Isorhamnetin 3-O-xyloside	0.317	0.317	0.412 *
TFC	0.860 ***	0.860 ***	0.921 ***
TPC	0.418 *	0.418 *	0.713 **
MAC	0.959 **	0.959 **	0.780 **

*** significant *p* < 0.001; ** significant *p* < 0.01; * significant *p* < 0.05.

## Data Availability

All the data supporting the findings of this study are included in this article.

## Supplementary Materials

The following supporting information can be downloaded at: https://www.mdpi.com/article/10.3390/molecules27113590/s1, Figure S1. Loading scores of the component 1 and component 2 of the PCA (presented in Figure 5) linking the phytochemical profile and antioxidant capacity of the stamen and perianth extracts of 13 *N. lotus* populations originating from various floristic regions from Thailand. Figure S2. Calibration curves for A. TPC (total phenolic content), B. TFC (total flavonoid content), and C. MAC (total monomeric anthocyanin content); Table S1. HPLC quantification of the main flavonoids in the stamen (A) and perianth (B) extracts of the 13 *N. lotus* populations from their natural habitats covering all the floristic regions in Thailand.

## Sample Availability

Samples of the compounds are available from the authors.
