# Supplementary material for: Differential Flavonoid and Other Phenolic Accumulations and Antioxidant Activities of Nymphaea lotus L. Populations throughout Thailand
Source: Molecules. 2022 Jun 2;27(11):3590. doi: 10.3390/molecules27113590 (PMC9182519; doi:10.3390/molecules27113590)
Supplement: Supplementary file 1 [file molecules-27-03590-s001.zip › molecules-1716756-supplementary.pdf]

Supplementary Materials

Differential Flavonoid and Other Phenolic Accumulations and Antioxidant Activities of *Nymphaea lotus* L. Populations Throughout Thailand

Duangjai Tungmunnithum [1,2,3,*], Samantha Drouet [2], Laurine Garros [2] and Christophe Hano [2,3,*]

[1] Department of Pharmaceutical Botany, Faculty of Pharmacy, Mahidol University, Bangkok 10400, Thailand
[2] Laboratoire de Biologie des Ligneux et des Grandes Cultures, INRAE USC1328, Campus Eure et Loir,
Orleans University, 28000 Chartres, France; samantha.drouet@univ-orleans.fr
[3] Le Studium Institue for Advanced Studies, 1 Rue Dupanloup, 45000 Orléans, France
* Correspondence: duangjai.tun@mahidol.ac.th (D.T.); hano@univ-orleans.fr (C.H.)

Contents

Figure S1. Loading scores of the component 1 and component 2 of the PCA (presented in Figure 5) linking the phytochemical profile and antioxidant capacity of the stamen and perianth extracts of 13 *N. lotus* populations originating from various floristic regions from Thailand.

Figure S2. Calibration curves for A. TPC (total phenolic content), B. TFC (total flavonoid content) and C. MAC (total monomeric anthocyanin content).

Table S1. HPLC quantification of the main flavonoids in the stamen (A) and perianth (B) extracts of the 13 *N. lotus* populations from their natural habitats cover the whole floristic regions in Thailand.

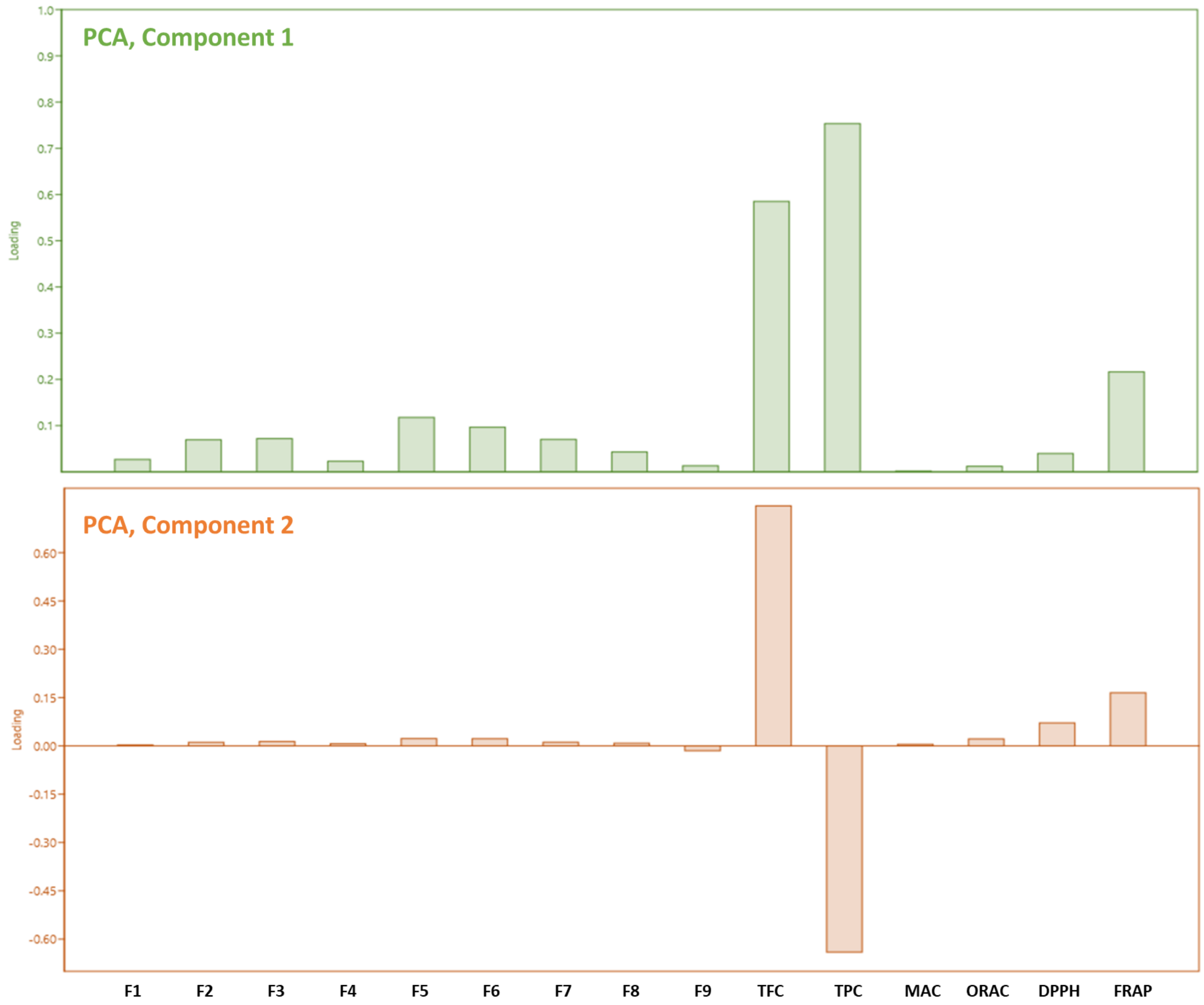

Figure S1. Loading scores of the component 1 and component 2 of the PCA (presented in Figure 5) linking the phytochemical profile and antioxidant capacity of the stamen and perianth extracts of 13 *N. lotus* populations originating from various floristic regions from Thailand. F1: Myricetin 3-O-galactoside; F2: Myricetin 3′-O-xyloside; F3: Quercetin 3-O-rhamnoside; F4: Chalcononaringenin 2′′-O-galactoside; F5: Kaempferol 3-O-galactoside; F6: Quercetin 3′-O-xyloside; F7: Isorhamnetin 7-O-galactoside; F8: Isorhamnetin 7-O-xyloside; F9: Isorhamnetin 3-O-xyloside; TFC: total flavonoid content; TPC: total phenolic content; MAC: monomeric anthocyanin content; ORAC, DPPH and FRAP: *in vitro* antioxidant assays.

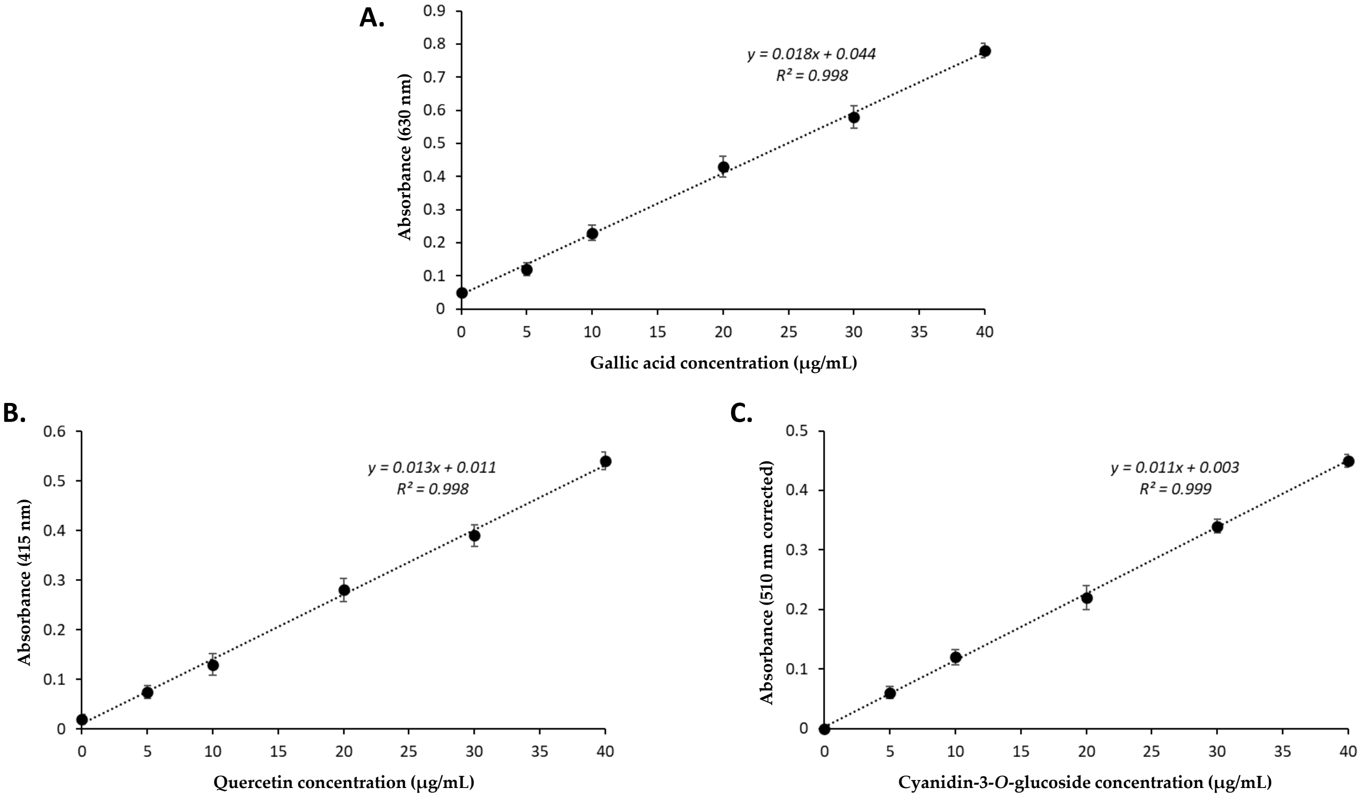

Figure S2. Calibration curves for A. TPC (total phenolic content), B. TFC (total flavonoid content) and C. MAC (total monomeric anthocyanin content).

Table S1. HPLC quantification of the main flavonoids in the stamen (A) and perianth (B) extracts of the 13 *N. lotus* populations from their natural habitats cover the whole floristic regions in Thailand.

Sample	F1		F2		F3		F4		F5		F6		F7		F8		F9	
	mean	SD	mean	SD	mean	SD	mean	SD	mean	SD	mean	SD	mean	SD	mean	SD	mean	SD
NLs#1	28.9	1.0	74.3	2.9	77.7	3.0	26.4	1.0	128.3	4.9	105.6	4.0	75.9	2.9	45.4	1.8	10.8	0.6
NLs#2	25.9	0.0	71.5	0.1	74.8	0.1	25.4	0.0	123.4	0.1	101.6	0.1	73.0	0.1	44.7	0.0	6.2	0.0
NLs#3	29.8	0.3	79.6	0.8	83.2	0.8	28.3	0.3	137.3	1.4	113.0	1.1	81.2	0.8	49.7	0.5	18.0	0.2
NLs#4	23.4	0.1	64.7	0.2	67.6	0.3	21.0	0.1	111.6	0.4	91.8	0.3	66.0	0.3	40.4	0.2	14.6	0.1
NLs#5	20.8	0.6	60.0	1.6	65.9	1.6	22.4	0.6	108.7	2.7	89.5	2.2	64.3	1.6	39.4	1.0	14.3	0.4
NLs#6	29.4	2.4	77.2	6.6	73.4	6.9	24.9	2.3	121.1	11.4	99.7	9.4	71.6	6.8	43.8	4.1	15.9	1.5
NLs#7	23.9	0.0	66.0	0.1	69.0	0.1	23.4	0.0	113.9	0.2	93.7	0.1	67.4	0.1	41.2	0.1	14.9	0.0
NLs#8	21.5	0.2	59.6	0.5	62.3	0.5	21.1	0.2	102.7	0.8	84.6	0.7	60.8	0.5	37.2	0.3	13.5	0.1
NLs#9	7.9	0.6	52.1	1.6	54.6	1.6	12.6	0.6	90.1	2.7	72.3	2.2	56.2	1.6	31.4	1.0	7.5	0.4
NLs#10	22.1	0.7	61.2	1.9	63.9	2.0	21.7	0.7	105.5	3.3	86.8	2.7	62.4	1.9	38.2	1.2	13.8	0.4
NLs#11	20.2	2.1	58.6	5.8	61.2	6.0	17.8	2.1	108.1	10.0	85.2	8.2	59.8	5.9	33.6	3.6	13.3	1.3
NLs#12	21.8	1.2	60.2	3.4	62.9	3.6	21.4	1.2	103.9	5.9	80.5	4.9	61.4	3.5	37.6	2.1	16.6	0.8
NLs#13	28.2	0.5	64.3	1.4	67.2	1.5	22.8	0.5	110.8	2.5	91.2	2.0	68.6	1.5	40.1	0.9	14.5	0.3
NLp#1	27.4	0.9	54.5	9.8	59.0	2.9	20.0	0.4	94.4	1.9	80.2	4.1	56.6	13.3	35.3	0.6	16.8	0.9
NLp#2	10.2	0.3	39.2	6.6	40.9	0.6	13.9	0.5	67.5	1.6	55.6	3.8	40.0	0.7	24.5	0.4	4.9	0.6
NLp#3	19.3	1.4	53.4	13.0	55.8	1.7	19.0	0.6	92.1	5.7	75.8	1.3	54.5	0.9	33.3	0.6	12.1	0.4
NLp#4	17.0	0.2	47.2	12.8	49.3	1.1	16.7	0.6	88.3	2.6	64.0	28.1	44.1	0.7	23.4	0.8	15.7	0.3
NLp#5	18.7	1.3	46.3	11.0	48.4	0.6	16.4	0.7	79.8	0.3	65.7	42.2	47.2	2.4	28.9	2.4	10.5	0.4
NLp#6	7.9	0.2	35.8	1.4	37.4	2.7	12.7	0.7	65.7	3.0	55.8	3.8	36.5	0.6	22.3	1.5	3.1	0.1
NLp#7	15.7	1.2	43.5	6.0	45.5	4.4	15.4	0.7	78.0	1.5	61.7	4.2	49.4	0.8	27.2	1.8	5.8	0.1
NLp#8	13.4	0.7	37.1	1.4	38.7	3.6	13.2	0.7	63.9	1.3	52.6	3.6	37.8	1.3	23.1	2.0	8.4	0.4
NLp#9	12.8	0.9	35.3	0.6	36.9	8.0	12.5	0.5	60.9	2.6	50.1	5.1	36.0	1.2	22.0	1.5	8.0	0.1
NLp#10	14.7	0.7	40.8	0.3	42.6	1.0	14.5	0.2	70.3	3.8	57.9	5.9	41.6	0.7	25.5	0.9	9.2	0.2
NLp#11	16.6	0.1	45.9	4.1	48.0	2.9	16.3	0.1	79.1	8.0	65.1	9.6	46.8	0.8	28.6	3.9	10.4	0.2
NLp#12	12.5	0.5	34.5	0.2	36.1	2.8	12.3	0.3	59.6	3.0	49.0	5.6	35.2	0.6	21.6	0.4	7.8	0.1
NLp#13	14.2	1.2	39.4	0.2	41.2	1.1	14.0	1.0	68.0	1.1	55.9	3.5	40.2	1.4	24.6	0.4	8.9	0.8

NL: Nymphea lotus *extract; s*: stamen; *p*: perianth; #i indicate the population number i; F1: Myricetin 3-O-galactoside; F2: Myricetin 3′-O-xyloside; F3: Quercetin 3-O-rhamnoside; F4: Chalcononaringenin 2′′-O-galactoside; F5: Kaempferol 3-O-galactoside; F6: Quercetin 3′-O-xyloside; F7: Isorhamnetin 7-O-galactoside; F8: Isorhamnetin 7-O-xyloside; F9: Isorhamnetin 3-O-xyloside. Means and standard deviations of 3 independent analysis.
